# Comparative circRNA Profiling in Human Erythroblasts Derived from Fetal Liver and Bone Marrow Hematopoietic Stem Cells Using Public RNA-Seq Data

**DOI:** 10.3390/ijms26178397

**Published:** 2025-08-29

**Authors:** Alawi Habara

**Affiliations:** Department of Biochemistry, College of Medicine, Imam Abdulrahman Bin Faisal University, P.O. Box 1982, Dammam 31441, Saudi Arabia; ahhabara@iau.edu.sa

**Keywords:** hemoglobin regulation, circular RNA, RNA-binding proteins, miRNA, erythropoiesis

## Abstract

Circular RNAs (circRNAs) are increasingly recognized as regulators of gene expression, although their roles in hematopoietic differentiation remain relatively understudied. This study compares circRNA expression profiles between erythroblasts derived from human fetal liver and bone marrow CD34^+^ hematopoietic stem cells using publicly available RNA-seq datasets (GEO: GSE90878). Twelve samples from each developmental source were analyzed. Differential expression analysis was performed, and circAtlas 3.0 was employed to predict interactions between circRNAs, microRNAs (miRNAs), and RNA-binding proteins. Differentially expressed miRNAs were curated from miRNA-seq data (GEO: GSE110936) profiling the same cell types. Principal component analysis of circRNA expression profiles demonstrated clear separation between erythroblasts from fetal liver and bone marrow, which was statistically confirmed by PERMANOVA (*p* = 0.001); though this effect size is small (R^2^ = 0.065). One circRNA, circALS2(4).1, was significantly upregulated in bone marrow-derived erythroblasts (adjusted *p* < 0.05), and ten additional circRNAs showed suggestive evidence for differential expression (adjusted *p* < 0.1). The resulting interaction networks reveal distinct circRNA landscapes and suggest regulatory circuits that may contribute to developmental differences in human erythropoiesis, indicating that the functions of circRNAs in hematopoietic development remain to be further elucidated.

## 1. Introduction

Erythropoiesis, the process of forming red blood cells, is crucial for oxygen delivery during fetal development and throughout adult life. During human embryogenesis, hematopoiesis occurs in multiple waves: starting from the yolk sac, followed by migration to the fetal liver, and finally localizing to the bone marrow, which remains the lifelong hematopoietic site after birth [1]. In the fetal liver, hematopoietic stem cells (HSCs) significantly expand due to a unique microenvironment that supports extensive proliferation, contrasting with the relatively quiescent state of adult bone marrow HSCs [1]. Specifically, fetal liver-derived CD34^+^ hematopoietic stem cells exhibit dramatic proliferation in vitro, reaching a 100-fold higher expansion capacity compared to adult bone marrow-derived CD34^+^ cells [2]. This distinct proliferative potential highlights fundamental differences in the regulatory mechanism between fetal and adult hematopoietic tissues.

Transcriptomic analyses comparing definitive erythropoiesis in fetal liver and adult bone marrow have identified several key molecular differences. For example, erythroid progenitor cells, burst-forming unit-erythroid (BFU-E), from fetal liver exhibit increased expression of genes associated with cell division and proliferation, and display distinct transcriptomic signatures compared to their bone marrow counterparts significantly higher expression of genes associated with cell cycle progression and proliferation, suggesting a robust cell division program to support rapid fetal growth [3]. Specifically, fetal liver erythroid progenitors display enhanced expression of key cell-cycle regulators such as glucocorticoid receptor (Nr3c1), Myc, and cyclin A2 (Ccna2), which promote rapid cell proliferation and expansion of the erythroid lineage during fetal development. During later stages of differentiation, terminal erythropoiesis, fetal liver erythroblasts upregulate biological processes associated with translation, indicating increased protein synthesis capacity. Moreover, pathways related to the tricarboxylic acid (TCA) cycle and hypoxia response are notably enriched, highlighting the metabolic adaptations of fetal erythroid cells necessary for efficient proliferation and hemoglobin production within the developing fetal liver environment. Additionally, fetal liver-derived erythroid cells predominantly express fetal and embryonic hemoglobin, in contrast to bone marrow-derived cells, which shift toward adult hemoglobin during maturation. This expression pattern is linked to decreased BCL11A, a key repressor of γ-globin, and elevated levels of LIN28B and IGF2BP1 in fetal liver erythroblasts, thereby sustaining high HbF production [2,4,5].

Despite these known differences at the transcriptomic and cellular level, the regulatory roles of non-coding RNAs, particularly circular RNAs (circRNAs), have not been systematically explored in fetal versus adult human erythropoiesis. Given the emerging evidence that circRNAs play significant roles in gene regulation and hematopoietic differentiation, exploring circRNA expression and associated regulatory networks in erythroblasts differentiated from fetal liver and adult bone marrow-derived CD34^+^ hematopoietic stem cells can provide important new insights into developmental erythropoiesis.

circRNAs are endogenous RNA molecules characterized by a covalently closed loop structure. They are primarily generated through a non-canonical splicing process known as backsplicing, in which a downstream splice donor site is joined to an upstream splice acceptor site, resulting in the formation of a circular RNA transcript [6,7]. Due to their closed-loop structure, circRNAs are more stable than linear RNAs, resisting degradation by exonucleases [7,8,9]. CircRNAs are generated through spliceosome-mediated backsplicing of exonic, intronic, or exon–intron sequences, a process influenced by specific RNA-binding proteins such as QKI, FUS, NF90/NF110, and ADAR, which can promote or inhibit their formation [6,7,8,10]. Functionally, circRNAs often act as miRNA sponges, binding and sequestering miRNAs to indirectly regulate gene expression [6,7]. Additionally, circRNAs interact with RBPs, contributing to post-transcriptional regulation, RNA stability, localization, and even facilitating circRNA translation into functional proteins when internal ribosome entry sites (IRES) are present [6,8]. circRNAs exhibit tissue-specific expression patterns and can be differentially regulated in various diseases, including cancers, neurological disorders, cardiovascular diseases, and inflammatory conditions [6,7]. For example, circRNAs such as circPVT1 and circHIPK3 have been implicated in leukemogenesis and other cancers, highlighting their potential as biomarkers or therapeutic targets [3]. Detection and quantification of circRNAs typically rely on RNA-sequencing methods using ribosomal RNA depletion strategies, complemented by bioinformatic algorithms to identify backsplice junctions, while experimental validation includes RNase R treatment and quantitative PCR (qPCR) with circRNA-specific primers [6,7].

CircAtlas 3.0 is a comprehensive resource containing more than three million curated circRNAs from 10 vertebrate species and 33 tissues. By integrating data from both Illumina and Nanopore sequencing platforms, CircAtlas 3.0 enables reconstruction of full-length circRNA isoforms and provides extensive functional annotations, including predicted miRNA- and RBP-binding sites, IRES/ORF predictions, and disease associations. This breadth of information makes CircAtlas 3.0 an essential tool for investigating circRNA biology and its clinical relevance [10,11].

Collectively, circRNAs represent a potential regulatory layer in hemoglobin and erythropoiesis regulation, with emerging roles across normal physiology and various diseases.

## 2. Results

### 2.1. circRNA Detection and Quantification

**Figure 1:**
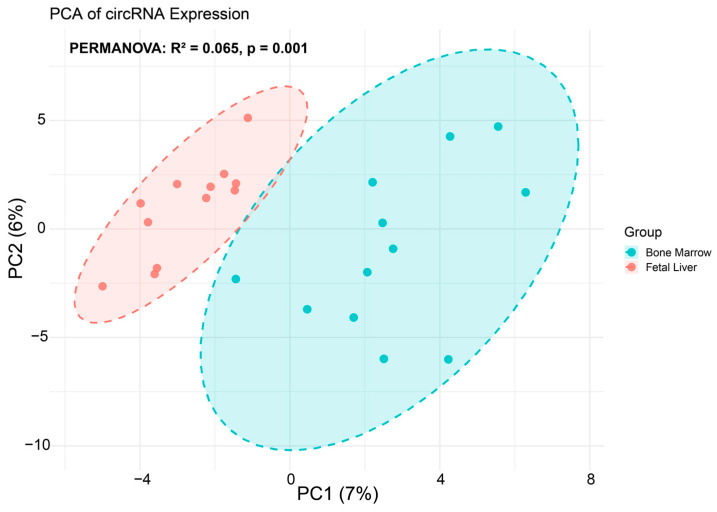
Principal component analysis (PCA) of circRNA expression profiles in human erythroblasts derived from fetal liver and bone marrow. Each point represents an individual sample, colored by origin (blue for bone marrow, red for fetal liver). PCA reveals a clear separation between the two groups with a low effective size. Statistical significance of group separation was confirmed by PERMANOVA (R^2^ = 0.065, *p* = 0.001).

Twelve samples from each developmental source were analyzed. The number of circRNAs detected per sample (≥2 supporting reads) was variable within each group, with erythroblasts derived from fetal liver CD34^+^ HSCs exhibiting a mean ± SD of 836 ± 300, and erythroblasts derived from bone marrow CD34^+^ HSCs exhibiting 695 ± 195 (Table S2). Statistical comparison revealed no significant difference between groups (Wilcoxon *p* = 0.29). Individual sample values are presented in Table S1, and the distribution across groups is illustrated in Figure S1. However, principal component analysis (PCA) of circRNA expression profiles revealed a statistically significant separation between erythroblasts derived from fetal liver and bone marrow, though the effect size was low (PERMANOVA R^2^ = 0.065, *p* = 0.001; Figure 1). This suggests that separation is marginal in terms of biological relevance in these samples.

### 2.2. Differentially Expressed circRNA Between Fetal Liver and Bone Marrow-Derived Erythroblasts

**Figure 2:**
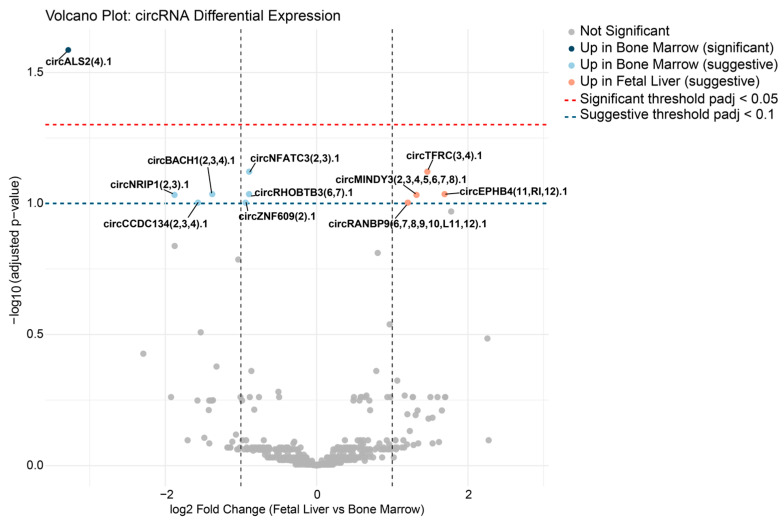
Volcano plot of circRNA differential expression between erythroblasts derived from fetal liver and bone marrow. Each point represents a circRNA, plotted by log_2_ fold change (fetal liver vs. bone marrow) and the negative log_10_ adjusted *p*-value. Significant circRNAs (adjusted *p* < 0.05) and suggestive circRNAs (adjusted *p* < 0.1) are indicated, with select circRNAs labeled. Upregulated circRNAs in each group are color-coded. Thresholds for significance and suggestiveness are indicated on the plot.

Differential expression analysis of circRNA profiles between erythroblasts derived from fetal liver and bone marrow CD34^+^ HSCs identified one circRNA, circALS2(4).1, as significantly upregulated in bone marrow-derived erythroblasts (adjusted *p* < 0.05). An additional ten circRNAs demonstrated highly suggestive evidence for differential expression (adjusted *p* < 0.1). Among the circRNAs demonstrating highly suggestive evidence for differential expression, four were upregulated in fetal liver-derived erythroblasts—namely, circMINDY3(2,3,4,5,6,7,8).1, circTFRC(3,4).1, circRANBP9(6,7,8,9,10,L11,12).1, and circEPHB4(11,RI,12).1. The remaining six suggestive circRNAs, circZNF609(2).1, circNFATC3(2,3).1, circNRIP1(2,3).1, circBACH1(2,3,4).1, circCCDC134(2,3,4).1, and circRHOBTB3(6,7).1, were upregulated in bone marrow-derived erythroblasts, as shown in Figure 2 and Table S3.

### 2.3. Predicted circRNA–miRNA Interaction Networks

**Figure 3:**
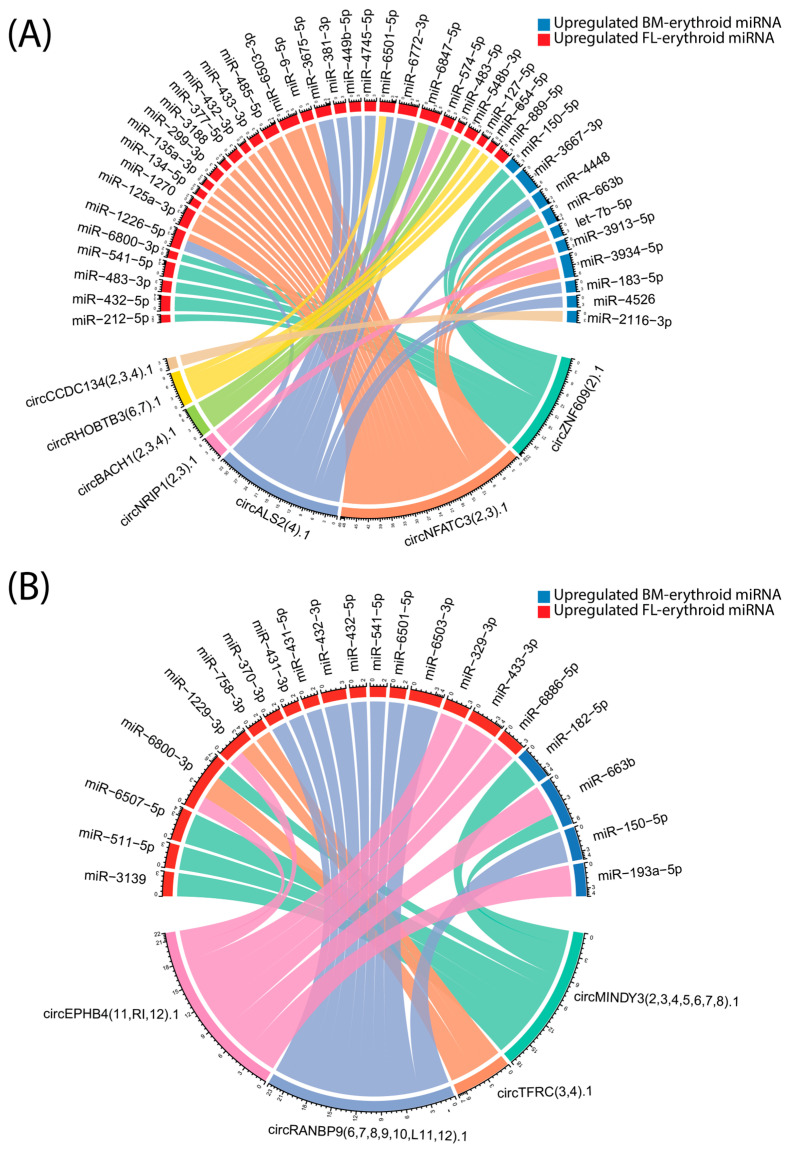
Chord plots of predicted circRNA–miRNA interactions in erythroblasts derived from bone marrow and fetal liver: (**A**) Predicted interactions between circRNAs upregulated in BM-derived erythroid cells and miRNAs upregulated in BM or FL erythroid cells. (**B**) Predicted interactions between circRNAs upregulated in FL-derived erythroid cells and miRNAs upregulated in BM or FL erythroid cells. CircRNAs and miRNAs are displayed as segments around the circle, with connecting lines indicating predicted binding events; line thickness corresponds to the predicted strength (total number of predicted binding sites between each circRNA and miRNA) of interaction. Color coding distinguishes miRNAs upregulated in BM and FL. Tick marks represent the total number of predicted binding sites. The predicted binding sites were obtained from circAtlas 3.0 [11].

Predicted interactions between circRNAs upregulated in bone marrow and fetal liver-derived erythroid cells and miRNAs upregulated in erythroid cells from both developmental origins reveal a complex regulatory network during human erythropoiesis. As visualized in the chord plot (Figure 3), circRNAs and miRNAs are represented as sectors around the circle, with links indicating predicted binding events and their thickness corresponding to the overall binding strength. The color coding distinguishes miRNAs according to their tissue of upregulation (red for FL, blue for BM). Notably, certain circRNAs upregulated in bone marrow-derived erythroid cells, such as circALS2 and circZNF609, interact with both bone marrow- and fetal liver-upregulated miRNAs, suggesting cross-regulation between developmental pathways, see Figure 3A. Similarly, fetal liver circRNAs predominantly interact with miRNAs preferentially expressed in fetal erythroid cells, see Figure 3B. All binding site predictions were obtained from circAtlas 3.0. The comprehensive list of predicted binding sites between upregulated circRNAs in BM-derived erythroid cells and miRNAs is provided in Table S4, while Table S5 presents the corresponding interactions for FL-derived circRNAs. Strength is defined as the total number of predicted binding sites across all three prediction models, while confidence reflects the number of independent prediction algorithms (PITA, miRanda, TargetScan) that identified at least one binding site for a given miRNA–circRNA pair. Together, these findings highlight distinct and developmentally regulated circRNA–miRNA interaction networks during human erythropoiesis.

### 2.4. Predicted circRNA–RBP Interaction Networks

**Figure 4:**
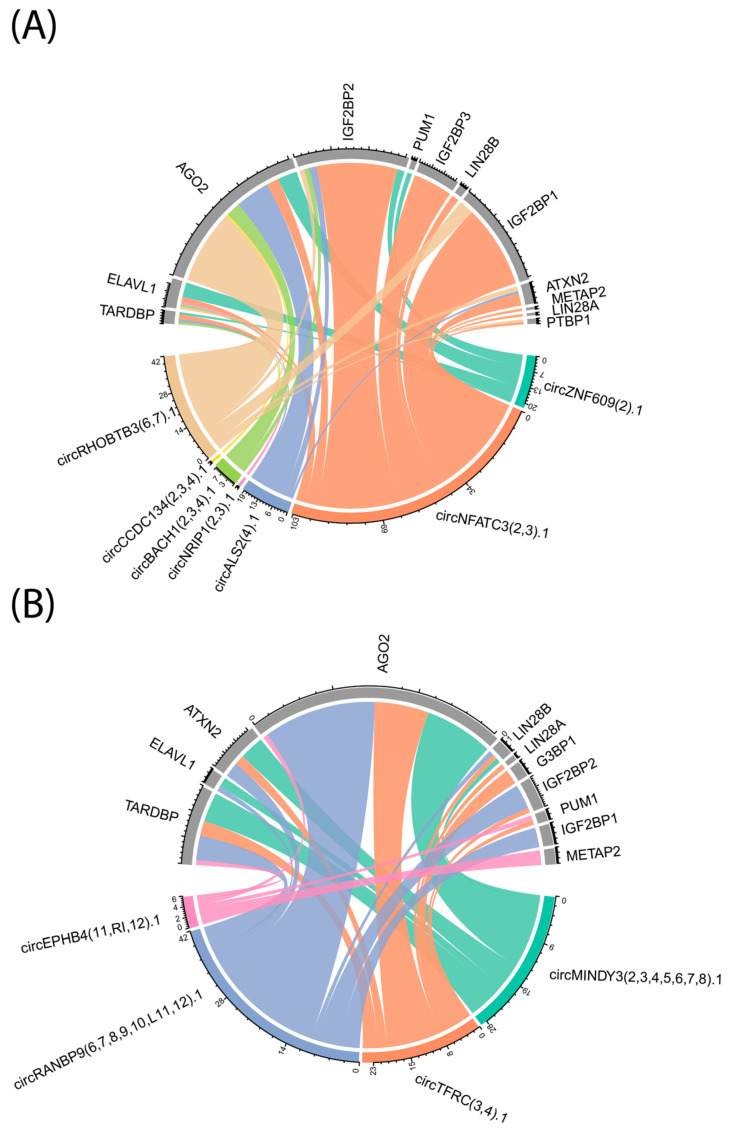
Chord plots of predicted interactions between upregulated circRNAs and key RNA-binding proteins in erythroid cells: (**A**) Predicted interactions between circRNAs upregulated in bone marrow (BM-derived erythroid cells and selected RBPs implicated in erythropoiesis or hemoglobin regulation). (**B**) Predicted interactions between circRNAs upregulated in fetal liver (FL)-derived erythroid cells and the same panel of RBPs. CircRNAs and RBPs are displayed as segments around the circle, with connecting lines indicating predicted binding events. Line thickness corresponds to the predicted strength of interaction, defined as the total number of predicted binding sites within circexons for each circRNA–RBP pair. The predicted binding sites were obtained from circAtlas 3.0 [11].

Predicted interactions between upregulated circRNAs and key RNA-binding proteins (RBPs) in erythroid cells were systematically analyzed using circAtlas 3.0, and the results are visualized in Figure 4. The chord plot illustrates the connectivity between circRNAs that are upregulated in bone marrow (BM)-derived erythroid cells (Figure 4A) or fetal liver (FL)-derived erythroid cells (Figure 4B) and a selected panel of RBPs previously implicated in erythropoiesis or hemoglobin regulation. Notably, several circRNAs demonstrate potential binding to multiple RBPs, and conversely, certain RBPs such as AGO2, IGF2BP1, IGF2BP2, and LIN28B exhibit extensive predicted interactions with several circRNAs in both BM- and FL-derived contexts. The total number of predicted binding sites for each circRNA–RBP pair, as indicated by the tick marks along each arc, varies widely, with some RBPs showing high binding site density and others with more limited interactions. These in silico findings highlight distinct and overlapping circRNA–RBP interaction networks across developmental origins, suggesting that specific circRNA–RBP pairs may contribute to the regulation of gene expression programs during erythroid differentiation. Table S6 summarizes the predicted interactions between upregulated circRNAs and key RNA-binding proteins in Erythroid Cells. Table S7 summarizes all the RBPs known to interact with the detected circRNA.

### 2.5. mRNA Expression for the RNA-Binding Protein in Fetal Liver and Bone Marrow-Derived Erythroblasts

**Figure 5:**
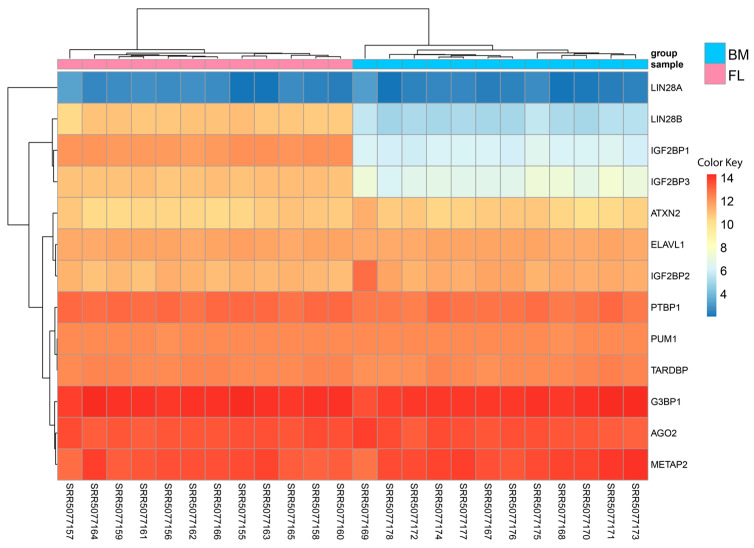
Expression heatmap for mRNA for selective RNA-binding proteins in fetal liver and bone marrow-derived erythroblasts. Raws represent selective RBPs; columns represent individual samples, with sample names on the x-axis. Samples are annotated by group (pink: FL, blue: BM) in the top bar. Expression values are shown as rlog-transformed counts, with higher expression indicated by warmer colors (red) and lower expression by cooler colors (blue), as illustrated by the color key.

To determine whether the RBPs identified in our circRNA interaction predictions are expressed in these cell types, we quantified the mRNA expression of each RBP in fetal liver and bone marrow-derived erythroblasts. Figure 5 shows the heatmap expression for these RBPs. All the selective RBPs were expressed in these cells. The mRNA expression for LIN28B, IGF2BP1, and IGF2BP3 is significantly higher in fetal liver-derived erythroblasts than in bone marrow-derived erythroblasts.

### 2.6. CircRNA–miRNA–(RBP)mRNA Network

**Figure 6:**
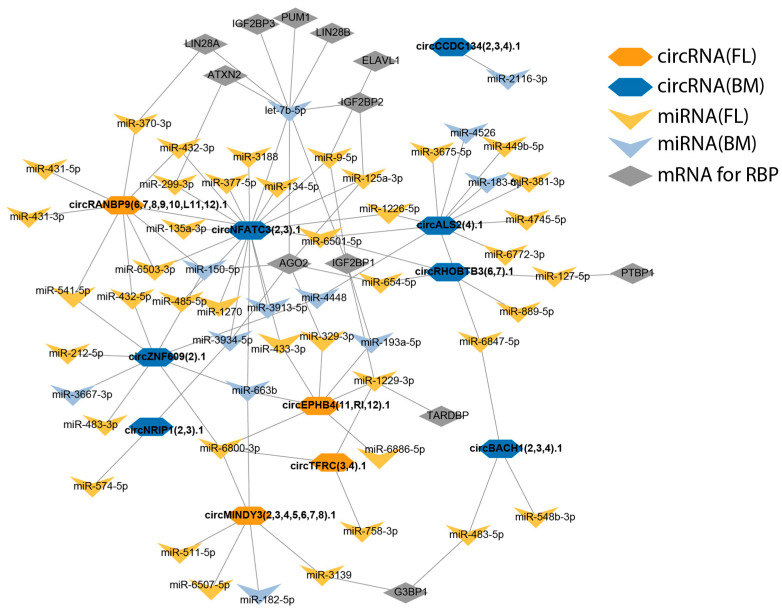
circRNA–miRNA–RBP(mRNA) integrative network. Orange hexagons indicate circRNAs derived from fetal liver (circRNA(FL)), blue hexagons represent circRNAs from bone marrow (circRNA(BM)). Yellow and light blue arrows correspond to miRNAs associated with fetal liver (miRNA(FL)) and bone marrow (miRNA(BM)), respectively. Gray rectangles indicate mRNAs encoding RNA-binding proteins (RBPs). Lines represent regulatory interactions.

To further understand the multilayered post-transcriptional regulatory landscape in erythropoiesis, an integrated circRNA–miRNA–(RBP)mRNA network was constructed (Figure 6). Interactions among differentially expressed circRNAs, miRNAs, and mRNAs encoding RBPs were mapped to uncover potential regulatory circuits that may coordinate gene expression during fetal liver and bone marrow erythroid differentiation. Through this integrative analysis, deeper insights into the interplay between non-coding and coding RNAs were gained, and candidate regulatory molecules or hubs that could play key roles in erythroid development and related disorders were highlighted.

## 3. Discussion

Despite the growing recognition of circRNAs as important regulators in various biological processes, relatively few studies have explored their roles in erythropoiesis and hemoglobin regulation, particularly during human development [10,12,13,14].

In β-thalassemia, transcriptome profiling has identified more than 2000 dysregulated circRNAs, many of which are linked to erythroid differentiation and globin gene regulation [13]. Among these, hsa_circRNA_100466, also known as hsa-LBR_0002 in circAtlas and circLBR(7,8).1 as its uniform ID [10], emerged as a key regulator of HbF through a circRNA-100466/miR-19b-3p/SOX6 axis, where SOX6 functions as a known repressor of γ-globin, thereby directly connecting circRNA activity to HbF silencing [13].

Beyond mechanistic networks, circRNAs also hold diagnostic potential. For instance, circ_0008102, also known as hsa-LCOR_0002 in circAtlas and circLCOR(5,6,7).1 as its uniform ID [10], was found to be significantly downregulated in the peripheral blood of β-thalassemia patients, with levels correlating to disease severity [13]. Diagnostic performance was evaluated for circ_0008102 expression in peripheral blood of pediatric β-thalassemia patients. circ_0008102 distinguished transfusion-independent patients from those requiring transfusion with an AUC of 0.733 (95% CI: 0.590–0.877; sensitivity 72.2%; specificity 68.6%; *p* = 0.006) [13]. Additionally, it differentiated transfusion-independent patients from healthy controls with an AUC of 0.711 (95% CI: 0.554–0.868; sensitivity 72.2%; specificity 69.0%; *p* = 0.016) [13]. These findings suggest that circ-0008102 could be a novel biomarker for identifying transfusion-independent pediatric β-thalassemia patients.

Furthermore, hsa_circ_0005245, also known as hsa-TBCD_0002 in circAtlas and circTBCD(18,19,20,21,22,23).1 as its uniform ID, has been experimentally validated to promote γ-globin expression by sponging miR-425-3p and thereby derepressing GATA2, a transcription factor essential for sustaining HbF production [14]. Overexpression of hsa_circ_0005245 in K562 shows a significant increase in HbF level, confirming that hsa_circ_0005245/hsa-miR-425-3p/GATA2 enhances γ-globin levels, providing functional evidence of circRNA-mediated regulation [14]. Collectively, these findings illustrate that circRNAs contribute both to the molecular control of γ-globin switching and to the pathophysiology of hemoglobinopathies, underscoring their significance as potential therapeutic targets and biomarkers in disorders of erythropoiesis.

Although these few recent studies have demonstrated that circRNAs can regulate γ-globin expression or serve as potential biomarkers [12,13,14], the available studies remain limited, and circRNA contributions to hemoglobin regulation and erythropoiesis are only beginning to be defined.

By contrast, most existing research in erythropoiesis has focused on linear transcripts, leaving the broader contribution of circRNAs to erythroid differentiation and globin gene regulation largely unexplored. To address this gap, the present study profiled circRNA expression and predicted interaction networks in erythroblasts derived from fetal liver and bone marrow hematopoietic stem cells, utilizing publicly available total RNA-seq data. While the overall numbers of circRNAs detected per sample were not significantly different between erythroblasts derived from fetal liver and those derived from bone marrow, PCA revealed statistically significant group separation based on circRNA expression profiles, underscoring the existence of distinct circRNA landscapes associated with each developmental origin; however, the effect size was small, indicating that the biological separation between these samples group is marginal. The circRNA–miRNA interaction network analysis highlighted both tissue-restricted and cross-regulatory relationships, with several circRNAs upregulated in bone marrow showing predicted interactions with miRNAs preferentially expressed in either bone marrow or fetal liver. For example, circALS2 and circZNF609 interact with both BM and FL upregulated miRNAs, suggesting a potential role in integrating developmental signals during erythroid maturation. The existence of such cross-regulation may reflect mechanisms that fine-tune gene expression transitions between fetal and adult erythropoiesis.

Predicted circRNA–RBP interaction networks revealed extensive binding potential for several RBPs with established roles in erythropoiesis and hemoglobin regulation, including AGO2, IGF2BP1, IGF2BP2, and LIN28B. Notably, certain circRNAs were predicted to bind multiple RBPs, while individual RBPs displayed broad connectivity with circRNAs across both developmental contexts. These networks suggest that circRNAs may serve as important modulators of post-transcriptional gene regulation during erythroid differentiation, either by sequestering RBPs or facilitating the formation of ribonucleoprotein complexes.

To evaluate whether the selected RBPs are expressed in our samples, mRNA expression analysis was performed, which confirmed that the corresponding mRNAs for these RBPs are indeed present. Notably, the mRNA expression levels of LIN28B, IGF2BP1, and IGF2BP3 were significantly higher in fetal liver-derived erythroblasts compared to bone marrow-derived erythroblasts. However, as circRNAs may directly bind RBPs, acting as molecular sponges or scaffolds to modulate RBP localization, stability, and function, this could not be further assessed due to the absence of proteomic data for these samples. Nevertheless, circRNAs can also regulate RBPs indirectly via miRNA–mRNA interaction networks, as illustrated in Figure 6.

Taken together, these findings demonstrate that circRNA–miRNA–(RBP)mRNA interaction networks are developmentally regulated, likely contributing to the distinct molecular identities observed between fetal and adult erythroid cells. The identification of specific circRNAs and their predicted interactions with regulatory miRNAs and RBPs suggests these molecules may serve as critical modulators in key developmental processes, including hemoglobin gene regulation.

However, several limitations should be considered when interpreting these results. First, the circRNA detection in this study was conducted using total RNA-seq libraries rather than circRNA-enriched preparations. Consequently, this approach may have reduced sensitivity in detecting low-abundance or tissue-specific circRNAs, potentially omitting circRNAs that might have been identified with enrichment protocols. Second, all circRNA–miRNA and circRNA–RBP interactions were predicted in silico using computational tools, primarily circAtlas 3.0 and miRTarBase [11,15]. Thus, experimental validation is essential to confirm the biological relevance and specificity of these interactions.

Additionally, the current study did not perform co-expression analyses. Therefore, proposed regulatory roles for circRNAs upregulated in one tissue (e.g., bone marrow) that interact with miRNAs or RBPs upregulated in another tissue (e.g., fetal liver) remain speculative. Without experimental evidence, cross-tissue regulatory effects cannot be conclusively inferred from computational predictions alone. Finally, while selected RBPs with known roles in erythropoiesis and hemoglobin regulation were examined, the lack of comprehensive proteomic profiling of RBPs in erythroid cells derived from fetal liver and bone marrow limits a complete understanding of circRNA–RBP dynamics.

Despite these limitations, the findings underscore the potential role of circRNAs as regulatory elements in hemoglobin expression and erythroid differentiation, both in normal physiology and disease states. Systematic profiling and in silico analyses of circRNA expression and interactions in fetal liver and bone marrow-derived erythroblasts offer new insights into erythroid molecular genetics. These insights may facilitate the discovery of novel biomarkers or therapeutic targets for hemoglobinopathies and related erythroid disorders.

Future studies employing experimental validation methods, such as circRNA knockdown, overexpression, or pulldown assays, will be critical for establishing the mechanistic significance of the predicted interactions. Such work may identify promising targets for therapeutic modulation of erythropoiesis or reactivation of fetal hemoglobin, offering potential advances in the treatment of hemoglobinopathies.

## 4. Materials and Methods

### 4.1. Data Acquisition and Quality Control

Publicly available total RNA-seq data were obtained from the Gene Expression Omnibus (GEO: GSE90878). In the original study, human fetal liver and adult bone marrow CD34+ hematopoietic stem and progenitor cells (HSPCs) were cultured and differentiated into erythroblasts as described in the original publication [16]. Total RNA was extracted using the miRNEASY kit (Qiagen, Hilden, Germany), and stranded paired-end (100 bp) RNA sequencing was performed on the Illumina HiSeq 2000 platform [16,17].

For the present analysis, raw sequencing data were downloaded and subjected to quality assessment using FastQC (v0.11.9). Adapter sequences and low-quality bases were trimmed using Trimmomatic (v0.39) prior to downstream analysis. All samples demonstrated high-quality scores (Phred > 30), balanced nucleotide composition, and no adapter contamination.

### 4.2. CircRNA Detection and Annotation

Circular RNAs (circRNAs) were identified by aligning reads to the human reference genome (GRCh38, Gencode v39 annotation) using the STAR aligner (v2.7.11b) with chimeric junction detection enabled. Candidate circRNAs were parsed and annotated using CIRCexplorer2 (v2.3.8), employing the Gencode-derived refFlat gene annotation.

### 4.3. Quantification and Differential Analysis

Annotated circRNAs were filtered to retain candidates supported by at least two junction reads. Differential expression analysis was conducted using DESeq2 (v1.46.0) within RStudio (v4.4.2). Data management and preprocessing utilized tidyverse packages, while data visualization was performed with ggplot2 (v3.5.2). Statistical significance was evaluated through non-parametric tests, and group-level differences were analyzed using PERMANOVA.

### 4.4. mRNA Expression Analysis

Total RNA-seq FASTQ files, after quality control and trimming, were aligned to the human reference genome (GRCh38, Gencode v39) using STAR with default parameters for paired-end reads. Gene-level quantification was performed with featureCounts (v2.0.6) using the Gencode v39 annotation. The resulting raw count matrices were imported into RStudio, and Ensembl gene IDs were mapped to HGNC gene symbols using the biomaRt package. For differential expression analysis, genes with low counts across all samples were filtered out, and DESeq2 was used to identify genes differentially expressed between fetal liver and bone marrow groups. Fold changes and adjusted *p*-values were calculated for all remaining genes.

### 4.5. Data Acquisition Limitation

Total RNA was used for sequencing in the original study, without specific enrichment for circular RNAs. In this work, the raw total RNA-seq data generated by the original investigators were downloaded and analyzed for circRNA detection. Although the absence of circRNA enrichment may reduce sensitivity for some circRNAs, the significant candidates identified here likely represent robust and biologically relevant transcripts. With dedicated circRNA enrichment, these and additional circRNAs might be detected at even higher significance.

### 4.6. Identification of Erythropoiesis-Related miRNAs for circRNA Interaction Analysis

To characterize circRNA–miRNA interactions during human erythroid differentiation, two curated miRNA sets were derived from a published high-throughput sequencing study comparing fetal liver and bone marrow-derived erythroblasts [18]. Both the published lists of differentially expressed miRNAs (GEO accession GSE110936) were systematically analyzed to identify miRNAs specifically upregulated in fetal liver and bone marrow-derived erythroblasts. This approach ensured that downstream circRNA analyses were grounded in robust, biologically relevant miRNA profiles.

### 4.7. miRNAs Upregulated in Fetal Liver-Derived Erythroid Cells

A comprehensive set of miRNAs was identified from a published differential expression analysis comparing fetal liver and adult (bone marrow)-derived erythroblasts [18]. miRNAs with significant upregulation (adjusted *p*-value < 0.05 and positive log2 fold change) in fetal liver-derived erythroid cells were extracted and included in downstream analysis.

### 4.8. miRNAs Upregulated in Bone Marrow-Derived Erythroid Cells

Similarly, miRNAs upregulated (adjusted *p*-value < 0.05 and negative log2 fold change) in bone marrow-derived erythroid cells were curated from the same dataset.

### 4.9. Integrated circRNA Analysis

The curated miRNA lists were used to filter the predicted circRNA–miRNA interactions generated in circAtlas 3.0 [11], which utilizes in silico tools such as PITA, miRanda, and TargetScan. This strategy enabled a focused analysis of biologically relevant circRNA–miRNA pairs with potential functional roles during human erythropoiesis, allowing for a direct comparison between fetal liver and bone marrow developmental contexts.

Additionally, circAtlas 3.0 was used to predict circRNA–RBP interactions in fetal liver-derived erythroid cells. The results were then filtered to emphasize RBPs known to contribute to erythropoiesis and hemoglobin regulation [10]. To date, no studies have systematically compared the total RBP profiles between bone marrow- and fetal liver-derived erythroid cells. This lack of comparative data represents a limitation of the current analysis, as it restricts comprehensive interpretation of developmental differences in RBP–circRNA interactions.

Furthermore, the miRNA target for the RBP mRNA was obtained from miRTarBase, an experimentally validated miRNA–mRNA interactions database [15]. These data were subsequently used to construct the integrated circRNA–miRNA–mRNA regulatory network seen in Figure 6.

## 5. Conclusions

In summary, this study provides a comparative analysis of circRNA expression and predicted regulatory networks, including circRNA–miRNA and circRNA–RBP interactions, in erythroblasts derived from human fetal liver and bone marrow. One circRNA, circALS2(4).1, was found to be significantly upregulated in bone marrow-derived erythroblasts, with ten additional circRNAs showing suggestive developmental regulation. While the overall separation between developmental origins was statistically significant but modest in effect size, distinct interaction networks involving circRNAs, miRNAs, and RBPs were uncovered. These findings highlight a potential novel regulatory circuit that may contribute to developmental differences in human erythropoiesis.

Although the physiological roles of the identified circRNAs, their associated miRNAs, and RBPs remain to be established, the results suggest their potential involvement in regulating erythroid differentiation and hemoglobin expression. Future studies should focus on experimental validation of key circRNA candidates, such as circALS2(4).1, and their interactions with relevant miRNAs and RBPs, using approaches such as knockdown, overexpression, or pulldown assays. This will be essential to elucidate the specific functions and regulatory mechanisms of these molecules in erythropoiesis, and may identify new therapeutic targets for red blood cell disorders and hemoglobinopathies.

## Data Availability

All data analyzed in this study are publicly available from the Gene Expression Omnibus (GEO) under accession numbers GSE90878 and GSE110936. The datasets can be accessed at https://www.ncbi.nlm.nih.gov/geo/ (accessed on 10 February 2025) using the provided accession numbers.

## Supplementary Materials

The following supporting information can be downloaded at: https://www.mdpi.com/article/10.3390/ijms26178397/s1.

## Abbreviations

The following abbreviations are used in this manuscript:

circRNA	Circular RNA
miRNA	MicroRNA
RBP	RNA-binding protein
HSC	Hematopoietic stem cell
HSPC	Hematopoietic stem and progenitor cell
FL	Fetal liver
BM	Bone marrow
BFU-E	Burst-forming unit-erythroid
PCA	Principal component analysis
PERMANOVA	Permutational multivariate analysis of variance
RNA-seq	RNA sequencing
qPCR	Quantitative polymerase chain reaction
IRES	Internal ribosome entry site
ORF	Open reading frame
TCA cycle	Tricarboxylic acid cycle
SD	Standard deviation
GEO	Gene Expression Omnibus
DESeq2	Differential Expression Sequencing 2 (R/Bioconductor package for differential expression analysis)
RNase R	Ribonuclease R
HbF	Fetal hemoglobin
