# Supplementary material for: Comparative circRNA Profiling in Human Erythroblasts Derived from Fetal Liver and Bone Marrow Hematopoietic Stem Cells Using Public RNA-Seq Data
Source: Int J Mol Sci. 2025 Aug 29;26(17):8397. doi: 10.3390/ijms26178397 (PMC12428317; doi:10.3390/ijms26178397)
Supplement: Supplementary file 1 [file ijms-26-08397-s001.zip › Table S1 and S2.pdf]

Table S1: circRNA count per sample by group

Sample ID	circRNA count (≥2 reads)	Group*
SRR5077155	444	Fetal liver
SRR5077156	850	Fetal liver
SRR5077157	428	Fetal liver
SRR5077158	835	Fetal liver
SRR5077159	874	Fetal liver
SRR5077160	585	Fetal liver
SRR5077161	1371	Fetal liver
SRR5077162	1202	Fetal liver
SRR5077163	701	Fetal liver
SRR5077164	791	Fetal liver
SRR5077165	724	Fetal liver
SRR5077166	1226	Fetal liver
SRR5077167	515	Bone marrow
SRR5077168	438	Bone marrow
SRR5077169	754	Bone marrow
SRR5077170	597	Bone marrow
SRR5077171	490	Bone marrow
SRR5077172	608	Bone marrow
SRR5077173	934	Bone marrow
SRR5077174	1021	Bone marrow
SRR5077175	576	Bone marrow
SRR5077176	676	Bone marrow
SRR5077177	763	Bone marrow
SRR5077178	966	Bone marrow

*Sample group assignment: “Fetal Liver” = erythroblasts differentiated from human fetal liver $CD34^+$ hematopoietic stem cells; “Bone Marrow” = erythroblasts differentiated from human bone marrow $CD34^+$ hematopoietic stem cells.

Table S2: Summary Statistics for circRNAs Detected per Group

Group	Mean CircRNAs	SD CircRNAs	Min CircRNAs	Max CircRNAs
Bone Marrow	695	195	438	1021
Fetal Liver	836	300	428	1371
Wilcoxon p-value	0.29			
