# Supplementary material for: Comparative circRNA Profiling in Human Erythroblasts Derived from Fetal Liver and Bone Marrow Hematopoietic Stem Cells Using Public RNA-Seq Data
Source: Int J Mol Sci. 2025 Aug 29;26(17):8397. doi: 10.3390/ijms26178397 (PMC12428317; doi:10.3390/ijms26178397)
Supplement: Supplementary file 1 [file ijms-26-08397-s001.zip › Table S3.pdf]

Table S3: Top Differentially Expressed circRNAs Between Fetal Liver and Bone Marrow Erythroblasts

circAltas ID	Uniform ID	Gene Name	baseMean	log2FoldChange	lfcSE	stat	pvalue	padj
hsa-FAM188A_0003	circMINDY3(2,3,4,5,6,7,8).1	MINDY3	3.06	1.32	0.42	3.16	0.00157	0.09
hsa-ZNF609_0001	circZNF609(2).1	ZNF609	5.47	-0.94	0.31	-3.04	0.00233	0.10
hsa-NFATC3_0001	circNFATC3(2,3).1	NFATC3	7.72	-0.89	0.26	-3.49	0.00048	0.08
hsa-ALS2_0048	circALS2(4).1	ALS2	1.40	-3.28	0.81	-4.03	0.00006	0.03
hsa-NRIP1_0002	circNRIP1(2,3).1	NRIP1	2.13	-1.88	0.59	-3.16	0.00158	0.09
hsa-BACH1_0001	circBACH1(2,3,4).1	BACH1	3.31	-1.38	0.42	-3.25	0.00117	0.09
hsa-CCDC134_0001	circCCDC134(2,3,4).1	CCDC134	2.51	-1.57	0.51	-3.05	0.00229	0.10
hsa-TFRC_0013	circTFRC(3,4).1	TFRC	3.37	1.46	0.42	3.49	0.00048	0.08
hsa-RHOBTB3_0011	circRHOBTB3(6,7).1	RHOBTB3	7.47	-0.89	0.27	-3.35	0.00081	0.09
hsa-RANBP9_0001	circRANBP9(6,7,8,9,10,L11,12).1	RANBP9	3.71	1.21	0.39	3.10	0.00195	0.10
hsa-EPHB4_0003	circEPHB4(11,RI,12).1	EPHB4	2.35	1.69	0.52	3.24	0.00118	0.09

baseMean: Average expression level across all samples. log2FoldChange: Log2-transformed fold change between two conditions. lfcSE: log2 fold change of standard error. Stat: Statistical test value for differential expression. pvalue: Raw p-value from the statistical test. padj: Adjusted p-value (corrected for multiple testing).
