# Supplementary material for: Comparative circRNA Profiling in Human Erythroblasts Derived from Fetal Liver and Bone Marrow Hematopoietic Stem Cells Using Public RNA-Seq Data
Source: Int J Mol Sci. 2025 Aug 29;26(17):8397. doi: 10.3390/ijms26178397 (PMC12428317; doi:10.3390/ijms26178397)
Supplement: Supplementary file 1 [file ijms-26-08397-s001.zip › Table S4 Predicted Binding Sites between Upregulated circRNAs in BM-derived erythroid cells and miRNAs.pdf]

Table S4: Predicted Binding Sites between Upregulated circRNAs in BM-derived erythroid cells and miRNAs

circAtlas ID upregulated in erythroid cells derived from BM	miRNA name	miRNA group	# Binding sites by PITA	#Binding sites of miRanda	# Binding sites of targetScan	Strength	Confidence
circALS2(4).1	miR-1226-5p	FL	1	2	0	3	2
circALS2(4).1	miR-3675-5p	FL	1	2	1	4	3
circALS2(4).1	miR-381-3p	FL	1	2	0	3	2
circALS2(4).1	miR-449b-5p	FL	1	2	0	3	2
circALS2(4).1	miR-4745-5p	FL	1	2	0	3	2
circALS2(4).1	miR-6501-5p	FL	1	0	1	2	2
circALS2(4).1	miR-6772-3p	FL	1	2	2	5	3
circALS2(4).1	miR-6847-5p	FL	1	0	1	2	2
circALS2(4).1	miR-183-5p	BM	1	2	0	3	2
circALS2(4).1	miR-4448	BM	1	0	1	2	2
circALS2(4).1	miR-4526	BM	1	2	0	3	2
circBACH1(2,3,4).1	miR-483-5p	FL	1	0	1	2	2
circBACH1(2,3,4).1	miR-548b-3p	FL	0	2	1	3	2
circBACH1(2,3,4).1	miR-6847-5p	FL	1	2	0	3	2
circCCDC134(2,3,4).1	miR-2116-3p	BM	0	2	1	3	2
circNFATC3(2,3).1	miR-1226-5p	FL	1	0	1	2	2
circNFATC3(2,3).1	miR-125a-3p	FL	1	2	2	5	3
circNFATC3(2,3).1	miR-1270	FL	1	0	1	2	2
circNFATC3(2,3).1	miR-134-5p	FL	1	0	1	2	2
circNFATC3(2,3).1	miR-135a-3p	FL	1	0	1	2	2
circNFATC3(2,3).1	miR-299-3p	FL	1	0	2	3	2
circNFATC3(2,3).1	miR-3188	FL	1	0	1	2	2
circNFATC3(2,3).1	miR-377-5p	FL	1	2	0	3	2
circNFATC3(2,3).1	miR-432-3p	FL	1	0	1	2	2
circNFATC3(2,3).1	miR-433-3p	FL	1	2	0	3	2

circNFATC3(2,3).1	miR-485-5p	FL	1	2	1	4	3
circNFATC3(2,3).1	miR-6503-3p	FL	1	2	2	5	3
circNFATC3(2,3).1	miR-9-5p	FL	1	0	2	3	2
circNFATC3(2,3).1	let-7b-5p	BM	1	2	0	3	2
circNFATC3(2,3).1	miR-3913-5p	BM	1	2	0	3	2
circNFATC3(2,3).1	miR-3934-5p	BM	1	0	2	3	2
circNFATC3(2,3).1	miR-663b	BM	1	0	1	2	2
circNRIP1(2,3).1	miR-574-5p	FL	1	2	0	3	2
circNRIP1(2,3).1	miR-3934-5p	BM	1	2	0	3	2
circRHOBTB3(6,7).1	miR-127-5p	FL	1	0	1	2	2
circRHOBTB3(6,7).1	miR-6501-5p	FL	1	0	1	2	2
circRHOBTB3(6,7).1	miR-654-5p	FL	1	0	1	2	2
circRHOBTB3(6,7).1	miR-889-5p	FL	1	0	2	3	2
circZNF609(2).1	miR-212-5p	FL	1	0	1	2	2
circZNF609(2).1	miR-432-5p	FL	1	2	1	4	3
circZNF609(2).1	miR-483-3p	FL	1	2	0	3	2
circZNF609(2).1	miR-541-5p	FL	1	2	1	4	3
circZNF609(2).1	miR-6800-3p	FL	1	0	1	2	2
circZNF609(2).1	miR-150-5p	BM	1	0	2	3	2
circZNF609(2).1	miR-3667-3p	BM	1	2	3	6	3
circZNF609(2).1	miR-4448	BM	1	0	1	2	2
circZNF609(2).1	miR-663b	BM	1	0	1	2	2

FL= Fetal Liver; BM= Bone Marrow. Strength is the total of predicted binding sites across all predicted models. Confidence is the number of independent prediction algorithms that identified at least one binding site for a given miRNA–circRNA pair. All predictions were obtained from circAtlas 3.0.
