# Supplementary material for: Comparative circRNA Profiling in Human Erythroblasts Derived from Fetal Liver and Bone Marrow Hematopoietic Stem Cells Using Public RNA-Seq Data
Source: Int J Mol Sci. 2025 Aug 29;26(17):8397. doi: 10.3390/ijms26178397 (PMC12428317; doi:10.3390/ijms26178397)
Supplement: Supplementary file 1 [file ijms-26-08397-s001.zip › Table S5 Predicted Binding Sites between Upregulated circRNAs in FL-derived erythroid cells and miRNAs.pdf]

Table S5: Predicted Binding Sites between Upregulated circRNAs in FL-derived erythroid cells and miRNAs

circAtlas ID upregulated in erythroid cells derived from FL	microRNA name	miRNA group	# Binding sites by PITA	#Binding sites of miRanda	# Binding sites of targetScan	Strength	Confidence
circEPHB4(11,RI,12).1	FL	miR-1229-3p	1	0	1	2	2
circEPHB4(11,RI,12).1	FL	miR-329-3p	1	2	0	3	2
circEPHB4(11,RI,12).1	FL	miR-433-3p	1	2	1	4	3
circEPHB4(11,RI,12).1	FL	miR-6800-3p	1	0	1	2	2
circEPHB4(11,RI,12).1	FL	miR-6886-5p	0	2	1	3	2
circEPHB4(11,RI,12).1	BM	miR-193a-5p	0	2	2	4	2
circEPHB4(11,RI,12).1	BM	miR-663b	1	2	1	4	3
circMINDY3(2,3,4,5,6,7,8).1	FL	miR-3139	1	2	0	3	2
circMINDY3(2,3,4,5,6,7,8).1	FL	miR-511-5p	0	2	1	3	2
circMINDY3(2,3,4,5,6,7,8).1	FL	miR-6507-5p	0	2	2	4	2
circMINDY3(2,3,4,5,6,7,8).1	FL	miR-6800-3p	1	0	1	2	2
circMINDY3(2,3,4,5,6,7,8).1	BM	miR-182-5p	0	2	2	4	2
circMINDY3(2,3,4,5,6,7,8).1	BM	miR-663b	1	0	1	2	2
circRANBP9(6,7,8,9,10,L11,12).1	FL	miR-370-3p	1	0	1	2	2
circRANBP9(6,7,8,9,10,L11,12).1	FL	miR-431-3p	1	0	1	2	2
circRANBP9(6,7,8,9,10,L11,12).1	FL	miR-431-5p	1	0	1	2	2
circRANBP9(6,7,8,9,10,L11,12).1	FL	miR-432-3p	1	0	2	3	2
circRANBP9(6,7,8,9,10,L11,12).1	FL	miR-432-5p	1	0	1	2	2
circRANBP9(6,7,8,9,10,L11,12).1	FL	miR-541-5p	1	0	1	2	2
circRANBP9(6,7,8,9,10,L11,12).1	FL	miR-6501-5p	1	0	1	2	2
circRANBP9(6,7,8,9,10,L11,12).1	FL	miR-6503-3p	1	0	3	4	2
circRANBP9(6,7,8,9,10,L11,12).1	BM	miR-150-5p	0	2	2	4	2
circTFRC(3,4).1	FL	miR-1229-3p	1	0	1	2	2
circTFRC(3,4).1	FL	miR-6800-3p	1	2	0	3	2
circTFRC(3,4).1	FL	miR-758-3p	1	0	1	2	2

FL= Fetal Liver; BM= Bone Marrow. Strength is the total of predicted binding sites across all predicted models. Confidence is the number of independent prediction algorithms that identified at least one binding site for a given miRNA–circRNA pair. All predictions were obtained from circAtlas 3.0.
