# Supplementary material for: Comparative circRNA Profiling in Human Erythroblasts Derived from Fetal Liver and Bone Marrow Hematopoietic Stem Cells Using Public RNA-Seq Data
Source: Int J Mol Sci. 2025 Aug 29;26(17):8397. doi: 10.3390/ijms26178397 (PMC12428317; doi:10.3390/ijms26178397)
Supplement: Supplementary file 1 [file ijms-26-08397-s001.zip › Table S6 Predicted Interactions Between Upregulated circRNAs and Key RNA-Binding Proteins in Erythroid Cells.pdf]

Table S6: Predicted Interactions Between Upregulated circRNAs and Key RNA-Binding Proteins in Erythroid Cells

circAltas ID	RBP	Total binding sites in flanking sequences*	Total binding sites in cirexons*
circMINDY3(2,3,4,5,6,7,8).1	TARDBP	1	6
circMINDY3(2,3,4,5,6,7,8).1	ELAVL1	15	2
circMINDY3(2,3,4,5,6,7,8).1	ATXN2	0	5
circMINDY3(2,3,4,5,6,7,8).1	AGO2	4	14
circMINDY3(2,3,4,5,6,7,8).1	LIN28B	0	1
circZNF609(2).1	TARDBP	1	1
circZNF609(2).1	ELAVL1	3	6
circZNF609(2).1	AGO2	4	8
circZNF609(2).1	IGF2BP2	0	3
circZNF609(2).1	PUM1	0	2
circNFATC3(2,3).1	TARDBP	2	2
circNFATC3(2,3).1	IGF2BP2	0	33
circNFATC3(2,3).1	IGF2BP3	0	16
circNFATC3(2,3).1	LIN28B	0	3
circNFATC3(2,3).1	IGF2BP1	0	34
circNFATC3(2,3).1	ELAVL1	11	2
circNFATC3(2,3).1	ATXN2	0	5
circNFATC3(2,3).1	AGO2	0	5
circNFATC3(2,3).1	METAP2	0	1
circNFATC3(2,3).1	LIN28A	0	1
circNFATC3(2,3).1	PTBP1	9	1
circALS2(4).1	IGF2BP2	1	3
circALS2(4).1	AGO2	1	14
circALS2(4).1	ATXN2	7	1
circALS2(4).1	TARDBP	6	1
circNRIP1(2,3).1	ELAVL1	2	1
circBACH1(2,3,4).1	AGO2	0	6
circBACH1(2,3,4).1	ELAVL1	9	1
circBACH1(2,3,4).1	IGF2BP2	0	2
circBACH1(2,3,4).1	TARDBP	1	1
circCCDC134(2,3,4).1	AGO2	3	1
circTFRC(3,4).1	LIN28A	0	1
circTFRC(3,4).1	AGO2	7	11
circTFRC(3,4).1	TARDBP	1	3
circTFRC(3,4).1	G3BP1	0	3
circTFRC(3,4).1	IGF2BP2	0	1
circTFRC(3,4).1	PUM1	0	1
circTFRC(3,4).1	LIN28B	0	1
circTFRC(3,4).1	ATXN2	1	2
circRHOBTB3(6,7).1	IGF2BP2	0	2
circRHOBTB3(6,7).1	PTBP1	0	1
circRHOBTB3(6,7).1	AGO2	10	30

circRHOBTB3(6,7).1	IGF2BP1	0	6
circRHOBTB3(6,7).1	ATXN2	0	2
circRHOBTB3(6,7).1	ELAVL1	5	1
circRANBP9(6,7,8,9,10,L11,12).1	IGF2BP2	0	5
circRANBP9(6,7,8,9,10,L11,12).1	AGO2	15	23
circRANBP9(6,7,8,9,10,L11,12).1	TARDBP	1	5
circRANBP9(6,7,8,9,10,L11,12).1	ATXN2	1	3
circRANBP9(6,7,8,9,10,L11,12).1	IGF2BP1	0	4
circRANBP9(6,7,8,9,10,L11,12).1	ELAVL1	5	1
circRANBP9(6,7,8,9,10,L11,12).1	LIN28B	1	1
circEPHB4(11,RI,12).1	METAP2	0	3
circEPHB4(11,RI,12).1	AGO2	2	1
circEPHB4(11,RI,12).1	TARDBP	2	1
circEPHB4(11,RI,12).1	PUM1	0	1

*All predictions were obtained from circAtlas 3.0.
