# Supplementary figures and images for: Comparative circRNA Profiling in Human Erythroblasts Derived from Fetal Liver and Bone Marrow Hematopoietic Stem Cells Using Public RNA-Seq Data
Source: Int J Mol Sci. 2025 Aug 29;26(17):8397. doi: 10.3390/ijms26178397 (PMC12428317; doi:10.3390/ijms26178397)

# circRNA Detection per Sample by Group

Number of circRNAs ( $\geq 2$  reads)

1500

1000

500

0

ns

Bone Marrow

Fetal Liver

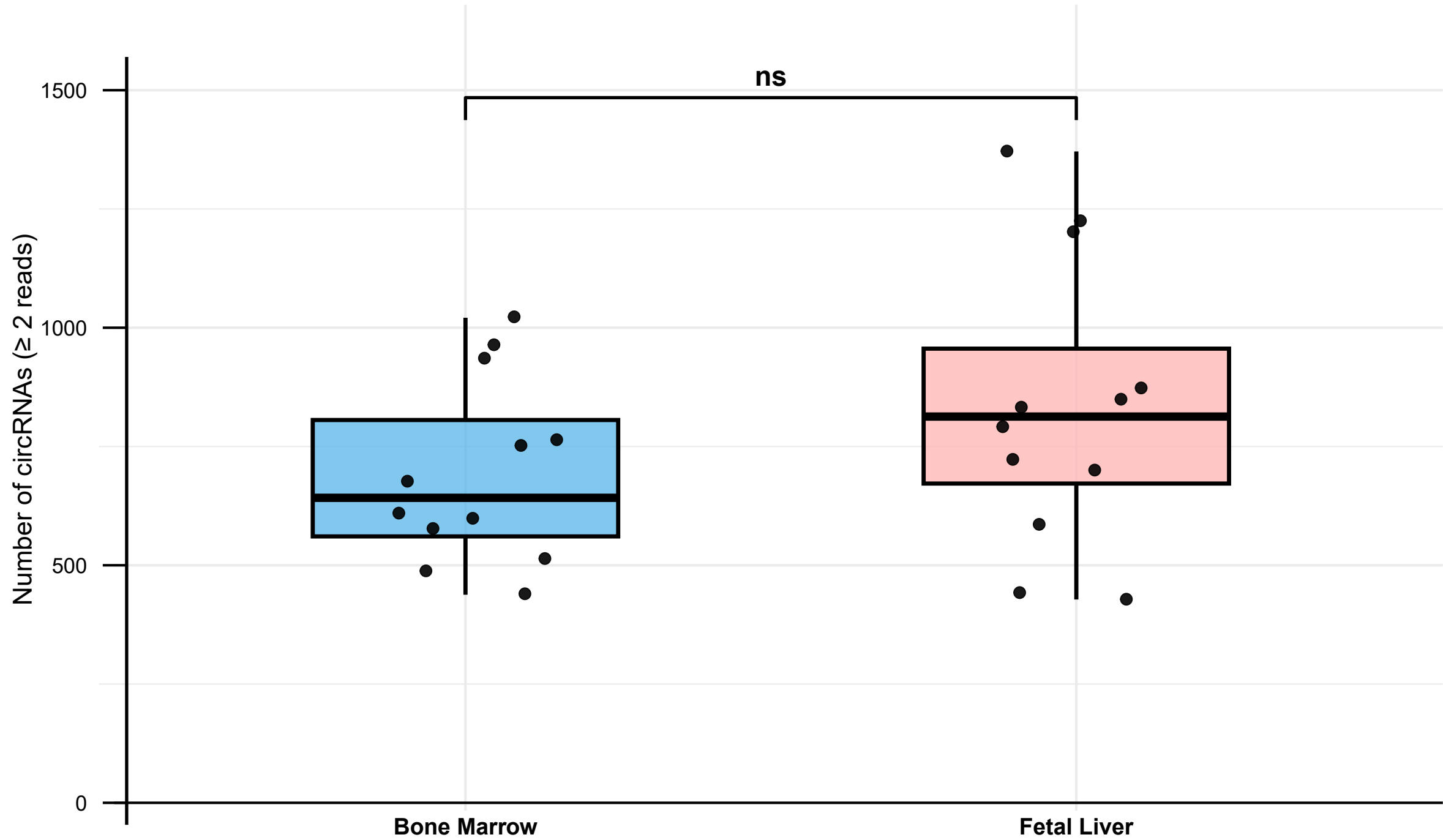

Supplement: Supplementary file 1 [file ijms-26-08397-s001.zip › Figure S1 circRNA number per sample by group.pdf]
